# A Scalable Lentiviral Workflow for Laboratory-Scale Generation of BCMA/GPRC5D Co-Transduced CAR-T Cells in Multiple Myeloma

**DOI:** 10.3390/cimb48070679

**Published:** 2026-06-30

**Authors:** Ewa Nowak, Emilia Morawiec, Adam Pudełko, Agnieszka Polak, Mateusz Broncel, Daria Matczyńska, Dawid Zamojski, Michał Czerwinski, Anna Bednarska-Czerwińska

**Affiliations:** 1 Gyncentrum Sp. z o.o. Medical Diagnostic Laboratory, Genetics and Molecular Biology in Sosnowiec, 40-851 Katowice, Poland; e.morawiec@gyncentrum.pl (E.M.); a.pudelko@gyncentrum.pl (A.P.); a.polak@gyncentrum.pl (A.P.); m.broncel@gyncentrum.pl (M.B.); d.matczynska@gyncentrum.pl (D.M.); d.zamojski@gyncentrum.pl (D.Z.); m.czerwinski@gyncentrum.pl (M.C.); a.czerwinska@gyncentrum.pl (A.B.-C.); 2 Department of Microbiology, Academy of Silesia, 40-555 Katowice, Poland; 3 Department of Immunology, Academy of Silesia, 40-555 Katowice, Poland; 4 Department of Internal Medicine and Clinical Pharmacology, Faculty of Medical Science in Katowice, Medical University of Silesia, 40-055 Katowice, Poland; 5 Department of Data Science and Engineering, Silesian University of Technology, 44-100 Gliwice, Poland; 6 American Medical Clinic, 40-851 Katowice, Poland; 7 Department of Gynecology and Obstetrics, Faculty of Medicine in Zabrze, Academy of Silesia in Katowice, 40-555 Katowice, Poland

**Keywords:** lentiviral vector production, CAR-T manufacturing, BCMA, GPRC5D, multiple myeloma, co-transduction, T-cell engineering, cell therapy

## Abstract

Efficient and reproducible lentiviral vector production and T-cell transduction remain important technical challenges in CAR-T (Chimeric Antigen Receptor T-cell) cell manufacturing. In this study, we optimized HEK293T transfection and primary T-cell transduction parameters for lentiviral CAR constructs targeting BCMA (B-cell maturation antigen) and GPRC5D (G-protein coupled receptor family C group 5 member D). Lipofectamine 3000 and TurboFectin 8.0 were compared across different seeding densities and reagent-to-DNA ratios, with vector yields quantified by qPCR (Quantitative Polymerase Chain Reaction) and p24 ELISA (Enzyme-linked Immunosorbent Assay). Lipofectamine 3000 consistently generated higher viral titers and transduction efficiencies, as reflected by a greater proportion of GFP-positive (Green Fluorescent Protein) cells than TurboFectin 8.0, reaching peak titers of 9.65 × 10^8^ copies/mL for the anti-GPRC5D and 5.33 × 10^8^ copies/mL for the anti-BCMA vectors. Under optimized conditions, transduction efficiencies reached 43.8% GFP^+^ cells for BCMA-CAR and approximately 13–14% GFP-positive transduced cells for the GPRC5D construct within the tested TU/mL range. Co-transduction experiments yielded approximately 62–66% GFP^+^ cells with detectable BCMA-binding and presumptive GPRC5D-CAR-expressing subpopulations identified based on GFP reporter expression. Immunophenotypic analysis demonstrated a relatively stable CD4/CD8 distribution (~65/35), enrichment of effector memory CD8^+^ cells, and expression of activation-associated markers. Collectively, these findings describe an optimized lentiviral transfection and transduction workflow that may support the further development of dual-targeting BCMA/GPRC5D CAR-T manufacturing strategies in research and early translational settings.

## 1. Introduction

Lentiviral vectors in CAR-T therapy: Lentiviral vectors (LVs) are the leading platform for stable delivery of chimeric antigen receptor (CAR) constructs into primary T cells and remain essential for current CAR-T manufacturing strategies [1]. Their high transduction efficiency, durable transgene expression, and preservation of T-cell viability have enabled the clinical success of multiple approved CAR-T therapies, including tisagenlecleucel, which utilizes an EF1α-driven (Elongation Factor 1-alpha) lentiviral construct [2]. However, CAR-T production still relies on complex and expensive cGMP-compliant (Current Good Manufacturing Practice) lentiviral manufacturing workflows, limiting scalability and broader clinical accessibility [3]. Beyond manufacturing challenges, CAR construct architecture critically affects transgene expression and T-cell fitness. In particular, the structural properties of the single-chain variable fragment (scFv) may influence CAR surface localization, tonic signaling, and cellular viability. Consequently, high transduction efficiency does not always correlate with effective CAR surface expression or functional persistence, as intracellular CAR retention and chronic signaling may promote T-cell exhaustion and apoptosis [2]. Together, these limitations highlight the need for optimized lentiviral engineering and transduction strategies capable of improving CAR expression, manufacturing efficiency, and therapeutic consistency in CAR-T cell production.

Lentiviral vectors (LVs) derived from HIV-1 (Human Immunodeficiency Virus type 1) remain one of the most widely used gene delivery systems in CAR-T cell therapy due to their ability to stably integrate transgenes into the host genome and efficiently transduce both dividing and non-dividing cells [4]. Modern lentiviral systems retain only the minimal viral elements required for vector production and integration, improving biosafety while maintaining high transduction efficiency [5]. Despite these advantages, lentiviral vectors present important limitations, including immune recognition, insertional mutagenesis, transgene silencing, and the theoretical risk of replication-competent lentivirus formation. Consequently, newer generations of self-inactivating lentiviral systems have been developed to enhance vector safety and reduce the proportion of HIV-derived genetic elements [6]. Although LVs remain the current standard for CAR-T engineering because of their robust and durable gene delivery, their production is still labor-intensive, expensive, and difficult to scale, emphasizing the need for further optimization of vector safety and manufacturing efficiency.

Despite the remarkable clinical efficacy of BCMA-directed CAR-T therapies in relapsed and refractory multiple myeloma, disease relapse remains a major limitation, frequently associated with antigen escape, heterogeneous BCMA expression, and progressive T-cell exhaustion. Recent studies have demonstrated that downregulation or loss of BCMA expression may contribute to resistance following anti-BCMA immunotherapies, highlighting the need for alternative or complementary targets capable of maintaining durable tumor control. Among emerging antigens, GPRC5D has gained particular attention due to its high expression on malignant plasma cells and limited distribution in normal tissues, making it a promising therapeutic target, especially in patients previously exposed to BCMA-directed therapies. Consequently, dual-targeting strategies directed simultaneously against BCMA and GPRC5D have emerged as a rational approach to reduce antigen escape and improve therapeutic durability [7,8,9]. However, scalable and reproducible manufacturing frameworks enabling the efficient generation of dual-targeting BCMA/GPRC5D CAR-T products remain insufficiently characterized, particularly in decentralized and academic manufacturing settings. The selection of BCMA and GPRC5D as target antigens was driven by their complementary expression patterns and their role in mitigating antigen escape in multiple myeloma. BCMA is a well-established target, but its loss or downregulation has been observed in relapsed patients. In contrast, GPRC5D expression is often retained despite BCMA loss, supporting its use as a complementary target. Importantly, their largely non-overlapping expression in normal tissues further supports the rationale for dual-targeting strategies aimed at improving therapeutic coverage while maintaining a favorable safety profile.

Chimeric antigen receptors (CARs) are synthetic receptors designed to redirect T-cell activity toward tumor-associated antigens. Their modular architecture critically determines therapeutic efficacy and safety. The extracellular single-chain variable fragment (scFv) governs antigen specificity and signaling strength, while the hinge and transmembrane regions regulate receptor stability and epitope accessibility [10,11,12]. The intracellular domain, composed of CD3ζ and co-stimulatory molecules such as CD28, 4-1BB, OX40, or ICOS, controls CAR-T proliferation, persistence, cytokine production, and exhaustion profiles [13]. Successive generations of CARs have been developed to improve therapeutic durability and antitumour activity. First-generation CARs containing only CD3ζ demonstrated limited persistence, whereas second-generation constructs incorporating a single co-stimulatory domain significantly enhanced T-cell expansion and survival. Third- and fourth-generation CARs further improved functionality through multiple co-stimulatory modules or inducible cytokine expression systems [14,15]. In this study, we presented an optimized lentiviral transfection and transduction strategy for generating CAR-T cells targeting BCMA and GPRC5D, two clinically relevant antigens in multiple myeloma. We additionally evaluated the influence of vector production and transduction parameters on functional CAR-T cell yield and expression efficiency.

The aim of this study was to optimize lentiviral vector production and CAR-T cell generation by evaluating key parameters affecting transfection and transduction efficiency. Specifically, we investigated the impact of carrier-to-plasmid DNA ratios and post-transfection viability of HEK293T packaging cells on lentiviral yield and GFP expression. Furthermore, we optimized the transduction of CD4+ and CD8+ T cells using CAR-encoding lentiviral vectors targeting the multiple myeloma-associated antigens BCMA and GPRC5D, including both single-target and co-transduction approaches.

## 2. Materials and Methods

### 2.1. 293T Cell Culture Conditions

The HEK293T cell line (European Collection of Cell Cultures [ECACC], Porton Down, Salisbury, UK; cat. no. 12022001) was used for lentiviral vector production due to its high transfectability and rapid proliferation. Cells were maintained in DMEM High Glucose supplemented with sodium pyruvate (Capricorn Scientific GmbH, Ebsdorfergrund, Germany; cat. no. DMEM-HPXA), 10% (*v*/*v*) heat-inactivated fetal bovine serum (BI Biological Industries Ltd., Beit HaEmek, Israel; cat. no. 04-127-1A), and L-glutamine (Capricorn Scientific GmbH, Ebsdorfergrund, Germany; cat. no. GLN-B). Cultures were grown in standard flasks (T25–T175), passaged at 70–80% confluence approximately every 48 h, and maintained at an optimal seeding density of 4 × 10^5^ cells/cm^2^. For passaging, cells were washed with PBS without magnesium and calcium (EURx Sp. z o.o., Gdańsk, Poland; cat. no. E0282-02) and detached using Accutase (Capricorn Scientific GmbH, Ebsdorfergrund, Germany; cat. no. ACC-1B). No inactivation step was required before subsequent cell processing. Cryopreservation was performed using FreezeMe One (Capricorn Scientific GmbH, Ebsdorfergrund, Germany; cat. no. FM1-F) at 3.5 × 10^6^–1 × 10^7^ cells/mL, with viability and density assessed using 0.4% Trypan Blue (PAN-Biotech GmbH, Aidenbach, Germany; cat. no. P08-341100) and the CellDrop Automated Cell Counter (DeNovix Inc., Wilmington, DE, USA).

### 2.2. CD4+ and CD8+ Cells Culture Conditions

Primary CD4^+^ (ATCC, Manassas, VA, USA; cat. no. PCS-800-016) and CD8+ (ATCC, Manassas, VA, USA; cat. no. PCS-800-017) T cells were used as effector populations with an approximate doubling time of ~40 h, depending on activation state and cytokine exposure. Cells were co-cultured at a 1:1 ratio in RPMI 1640 (ATCC, Manassas, VA, USA; cat. no. 30-2001) containing 2 mM L-glutamine and supplemented with 10% (*v*/*v*) FBS and TexMACS medium (Miltenyi Biotec B.V. & Co. KG, Bergisch Gladbach, Germany; cat. no. 130-097-196). Expansion and phenotype maintenance were supported by IL-2 (Interleukin 2) (Miltenyi Biotec B.V. & Co. KG, Bergisch Gladbach, Germany; cat. no. 130-097-742) (50 U/mL), IL-7 (Miltenyi Biotec B.V. & Co. KG, Bergisch Gladbach, Germany; cat. no. 130-093-937) (5 ng/mL), and IL-15 (Miltenyi Biotec B.V. & Co. KG, Bergisch Gladbach, Germany; cat. no. 130-093-955) (10 ng/mL). No antibiotics were used. Cultures were maintained in suspension and passaged every 48 h at 1 × 10^6^ cells/mL. All cultures were routinely tested and confirmed negative for mycoplasma contamination using the qPCR Mycoplasma Test Kit (PanReac AppliChem, Darmstadt, Germany; cat. no. A9019,0025).

### 2.3. Lentiviral Vectors

Two third-generation lentiviral CAR constructs with distinct co-stimulatory architectures were used. The anti-GPRC5D construct (ET150-8 scFv) incorporated ICOS-4-1BB-CD3ζ signaling domains (pCDCAR1) [8], whereas the anti-BCMA construct (25D2 scFv, single-chain variable fragment) contained CD28-OX40-CD3ζ domains (pCDCAR1) [16,17]. Both vectors included an EGFP reporter cassette for the identification of transfected and transduced cells. For transfection optimization, a GFP-expressing control plasmid (pLenti-EF1a-C-mGFP-P2A-Puro Lentiviral Gene Expression Vector, OriGene Technologies, Inc., Rockville, MD, USA; cat. no. PS100121) was used together with the Lenti-vpak Lentiviral Packaging Kit (OriGene Technologies, Inc., Rockville, MD, USA; cat. no. TR30037). Lentiviral particles were produced using a third-generation system consisting of transfer, packaging, and VSV-G envelope plasmids (CART-027CL-PLP1, CART-027CL-PLP2, CART-027CL-PLP-VSVG; OriGene Technologies, Inc., Rockville, MD, USA; cat. no. TR30037), ensuring high-titer production and improved biosafety.

### 2.4. Transfection of 293T Cells with Lentiviral Plasmids

**Figure 1:**
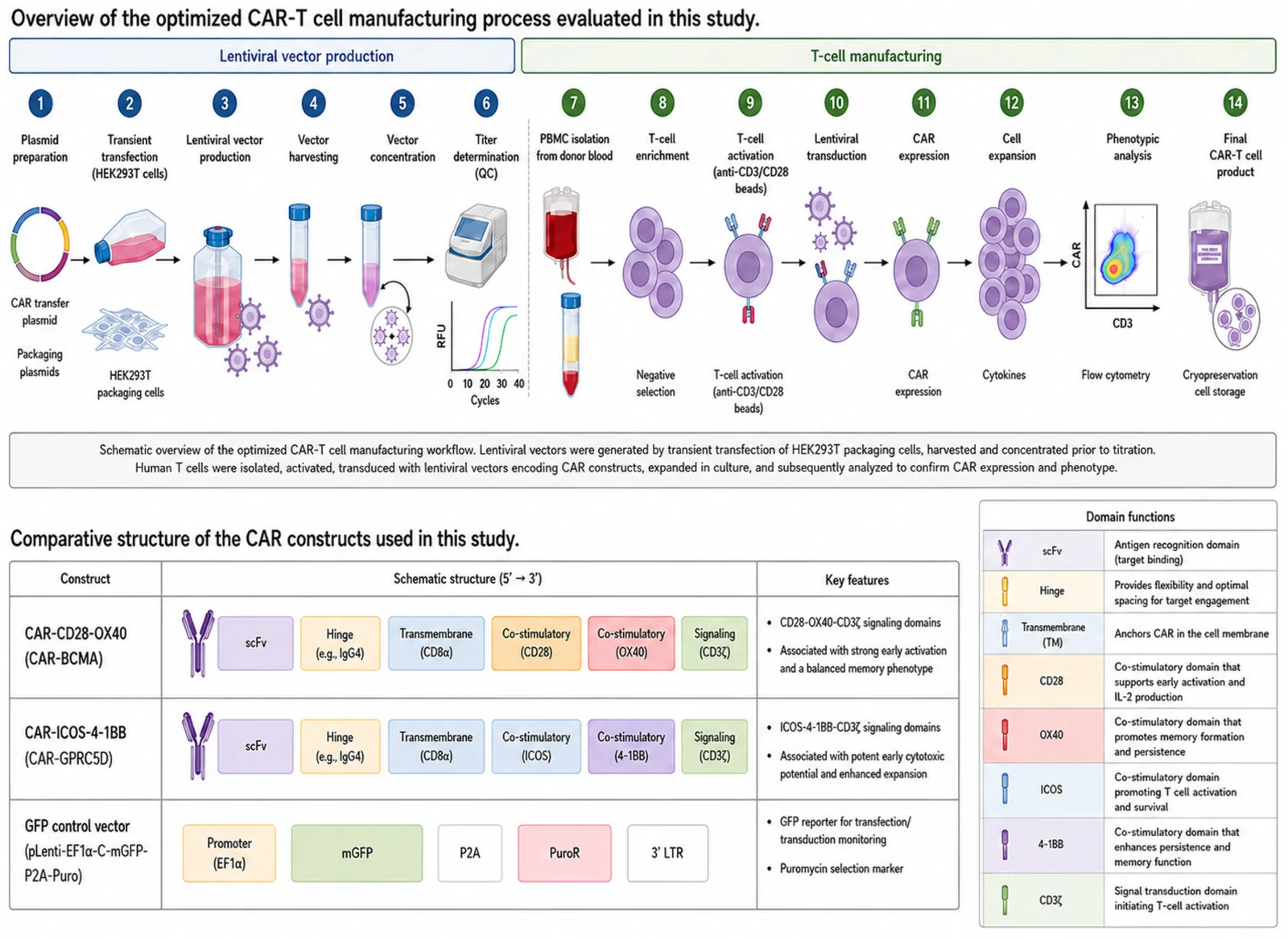
Schematic overview of lentiviral vector production, T-cell transduction, and CAR construct design. HEK293T cells were transfected with lentiviral transfer vectors encoding anti-BCMA CAR, anti-GPRC5D CAR, or GFP reporter constructs together with the lentiviral packaging system to generate lentiviral particles. Viral supernatants were harvested, concentrated, and titrated prior to transduction of activated human T lymphocytes. Following transduction, T cells were expanded in cytokine-supplemented culture medium and analyzed for transduction efficiency, viability, and immunophenotypic characteristics. The lower panel depicts the architecture of the CAR constructs used in this study, including the anti-BCMA CAR containing the CD28/OX40/CD3ζ signaling domains and the anti-GPRC5D CAR containing the ICOS/4-1BB/CD3ζ signaling domains, as well as the GFP reporter construct.

Efficient lentiviral production depended on both transfection chemistry and DNA-to-reagent ratios. A lipid-based system (Lipofectamine™ 3000, Invitrogen, Carlsbad, CA, USA; cat. no. L3000001) was compared with a polymer-based reagent (TurboFectin™ 8.0, OriGene Technologies, Inc., Rockville, MD, USA; cat. no. TF81001) [18]. Optimization was performed using a GFP reporter plasmid ((pLenti-EF1α-C-mGFP-P2A-Puro Lentiviral Gene Expression Vector, OriGene Technologies, Inc., Rockville, MD, USA; cat. no. PS100121) across multiple DNA-to-reagent ratios (Lipofectamine 3000: 1:1.5, 1:2.3, 1:3; TurboFectin 8.0: 1:2, 1:3) and seeding densities (5 × 10^4^–1.25 × 10^5^ cells/cm^2^), each in triplicate. The optimal condition (1 × 10^5^ cells/cm^2^, 1:3 ratio) was selected based on GFP expression (Axio Vert.A1 inverted microscope, Carl Zeiss Microscopy GmbH, Jena, Germany). These parameters were applied for the production of Anti-BCMA scFv(25D2) h (CD28-OX40-CD3ζ) CAR, pCDCAR1 and Anti-GPRC5D (ET150-8) h (ICOS-4-1BB-CD3ζ) CAR, pCDCAR1 lentiviral vectors. Detailed protocols are provided in Supplementary Materials S1–S3, including a scaled production workflow developed under patent application EPO 23461647.2. The scheme for lentiviral vector production is shown in Figure 1.

### 2.5. Concentration of Lentiviral Supernatants and Lentiviral Titer Determination

Lentiviral particles were concentrated using Lenti-X Concentrator (Takara Bio USA, Inc., San Jose, CA, USA; cat. no. 631231) at a 3:1 supernatant-to-reagent ratio, resuspended in 1/10 volume of PBS, incubated for 4 h at 4 °C, and stored at −80 °C. Viral titers were quantified after thawing using nucleic acid extraction (GeneMATRIX Viral RNA/DNA Purification Kit, EURx Sp. z o.o., Gdańsk, Poland; cat. no. E3592-02) followed by qRT-PCR with the Lenti-X™ qRT-PCR Titration Kit (Takara Bio USA, Inc., San Jose, CA, USA; cat. no. 631235) according to the manufacturer’s instructions. Results were validated by p24 antigen quantification (Lenti-X p24 Rapid Titer Kit, Takara Bio USA, Inc., San Jose, CA, USA; cat. no. 631476). Functional titers were reported as genome copies per milliliter (copies/mL) based on standard curve calibration. A comparison of qPCR- and p24-based quantification was used as complementary validation of lentiviral vector preparations, reflecting vector genome content and capsid protein levels, respectively. Differences between these readouts are expected due to the presence of non-functional or empty particles and the distinct nature of the measured analytes.

### 2.6. CD4^+^ and CD8^+^ Cell Transduction

Transduction and co-transduction of CD4^+^ and CD8^+^ T cells were optimized using two lentiviral vectors encoding Anti-BCMA scFv(25D2) h (CD28-OX40-CD3ζ) CAR, pCDCAR1 and Anti-GPRC5D (ET150-8) h (ICOS-4-1BB-CD3ζ) CAR, pCDCAR1. Functional viral titers (TU/mL) were established by serial dilution in co-cultured CD4^+^/CD8^+^ lymphocytes. On day 0, cells were seeded at 1 × 10^6^ cells/mL in TexMACS medium and activated with T Cell TransAct (Miltenyi Biotec B.V. & Co. KG, Bergisch Gladbach, Germany; cat. no. 130-128-758; 10 µL/mL). On day 1, cells were transduced with graded TU/mL of lentiviral vectors in the presence of Vectofusin-1 (Miltenyi Biotec B.V. & Co. KG, Bergisch Gladbach, Germany; cat. no. 130-111-163; 10 µL/mL). Spinoculation was performed at 400× *g* for 1 h at 32 °C, followed by incubation at 37 °C and 5% CO_2_ for 18 h. Following transduction, the medium was replaced with TexMACS supplemented with IL-7 (155 IU/mL) and IL-15 (290 IU/mL; Miltenyi Biotec). Cells were maintained at 1 × 10^6^ cells/mL with periodic scaling into larger culture vessels and medium exchange every 48 h. Immunophenotype and GFP expression were analyzed by flow cytometry on days 9, 11, and 14. The scheme for T-cell transduction, and CAR construct design is shown in Figure 1.

### 2.7. Immunophenotyping of T Lymphocytes After Lentiviral Transduction

Transduction efficiency was evaluated by GFP expression using flow cytometry (FITC channel; 488 nm excitation, 527/32 nm emission). CAR-T cell immunophenotyping was performed using antibodies against CD3 (APC-H7), CD8 (BV510), CD45RA (V-450), CD62L (PE-Cy7), CD25 (PE), and CD69 (BV-786) (Becton Dickinson BD Biosciences, San Jose, CA, USA). BCMA expression was detected using BCMA/TNFRSF17-APC (R&D Systems, Minneapolis, MN, USA; cat. no. FAB193A). Staining was performed in Stain Buffer (FBS) (BD Biosciences, San Jose, CA, USA; cat. no. 554656), with signal stabilization using Brilliant Stain Buffer (BD Biosciences, San Jose, CA, USA; cat. no. 566385/666514). Viability was assessed using propidium iodide. Data were acquired on a BD FACSMelody™ Cell Sorter (BD Biosciences, San Jose, CA, USA) and analyzed using BD FACSChorus™ software 1.1.20.0. Compensation was performed using BD™ CompBeads (BD Biosciences, San Jose, CA, USA; cat. no. 552843), FMO controls, or untransduced cells. Full staining details are provided in Supplementary Materials S4. This panel enabled identification of transduced GFP-positive populations and BCMA-binding CAR-expressing cells. Because direct detection of GPRC5D-CAR surface expression was not performed, GFP reporter expression was used as a surrogate marker of successful lentiviral transduction. Therefore, GFP^+^/BCMA-binding cells were interpreted as co-transduced populations rather than definitively confirmed dual-CAR-expressing cells.

### 2.8. Statistical Analysis

Due to the sample size and non-normal distribution of the data, variability was expressed as the median and interquartile range. For statistical analysis, the Kruskal–Wallis test was used. The post hoc Nemenyi test was used to determine which groups were significantly different. The statistical significance level was set at *p* < 0.05 (*). In addition, results with *p* values between 0.05 and 0.1 were considered to indicate non-significant trends (†). Statistical analysis was performed using R 4.4.0 (R Foundation for Statistical Computing, Vienna, Austria).

## 3. Results/Lentiviral Vector Production Optimization

### 3.1. Transfection of 293T Cells with Lentiviral Plasmids

Optimization of lentiviral vector production focused on identifying transfection conditions that maximized transgene expression while preserving HEK293T cell viability. We evaluated the influence of transfection reagent-to-DNA charge ratios and packaging cell density on GFP expression, viral yield, and post-transfection viability. Among the tested conditions, Lipofectamine 3000 consistently outperformed TurboFectin 8.0, achieving substantially higher percentages of viable GFP-positive cells and increased lentiviral copy numbers across multiple seeding densities. Although differences between conditions within the same reagent group were not statistically significant, comparative analysis revealed a clear trend favoring Lipofectamine 3000 over TurboFectin 8.0 (Figure S1, Supplementary Materials). Based on the combined assessment of transduction efficiency and cell viability, optimized conditions were selected for subsequent production of BCMA- and GPRC5D-targeting CAR lentiviral vectors. The optimized transfection parameters were subsequently applied to the production of lentiviral vectors encoding single- and dual-CAR constructs targeting the clinically relevant multiple myeloma antigens BCMA and GPRC5D, followed by downstream CD4+ and CD8+ T-cell transduction studies. A summary of the representative transfection results obtained with the GFP-encoding lentiviral plasmid is summarized in Table 1 (detailed data are presented in Table S1, Supplementary Materials), while transfection data for the Anti-BCMA scFv(25D2) h (CD28-OX40-CD3ζ) CART, pCDCAR1 and Anti-GPRC5D (ET150-8) h (ICOS-4-1BB-CD3ζ) CAR, pCDCAR1 constructs are presented in Table 2 as mean values from three independent experiments. After optimizing the transfection procedure with the GFP pLenti-EF1a-C-mGFP-P2A-Puro Lentiviral Gene Expression Vector and taking into account the parameter % of dead cells, the following condition was selected for further analyses: 10 × 10^4^ cells/cm^2^ transfected at a 1:3 ratio with Lipofectamine 3000 and Turbofectin 8.0 for Anti-BCMA scFv(25D2) h (CD28-OX40-CD3ζ) CART, pCDCAR1 and Anti-GPRC5D (ET150-8) h (ICOS-4-1BB-CD3ζ) CAR, pCDCAR1 plasmids. We did not observe statistically significant differences in the obtained results.

### 3.2. Lentiviral Titer Determination

Following large-scale transfection of HEK293T cells with Anti-BCMA scFv(25D2) h (CD28-OX40-CD3ζ) CART, pCDCAR1 and Anti-GPRC5D (ET150-8) h (ICOS-4-1BB-CD3ζ) CAR, pCDCAR1 lentiviral constructs, vector titers were quantified by qRT-PCR and independently validated by p24 ELISA (Figure S1). Concentrated supernatants demonstrated high lentiviral yields, with genomic titers of 5.33 × 10^8^ copies/mL for anti-BCMA and 9.65 × 10^8^ copies/mL for anti-GPRC5D vectors. These findings were confirmed by p24 quantification, which yielded titers of 6.91 × 10^8^/mL and 1.06 × 10^9^/mL, respectively (Table 3).

### 3.3. CD4^+^ and CD8^+^ Cells Transduction

Published data indicate that T-cell transduction efficiency is influenced by multiple variables beyond multiplicity of infection (MOI), particularly in primary lymphocytes, which are less permissive than adherent cell lines. Consequently, transduction enhancement strategies such as increased MOI, spinoculation, and chemical facilitators are often required. In this study, we systematically compared single-vector and dual-vector (co-transduction) strategies using lentiviral constructs encoding the anti-BCMA and anti-GPRC5D CARs. Functional titers (TU/mL), determined experimentally to standardize vector potency, were used to define transduction conditions (Tables S2 and S3, Supplementary Materials). The resulting titers were 2.34 × 10^6^ TU/mL for anti-BCMA and 1.71 × 10^6^ TU/mL for anti-GPRC5D vectors. In co-transduction settings, effective working titers ranged from 1.88 × 10^6^ to 3.89 × 10^6^ TU/mL for anti-BCMA and from 9.42 × 10^5^ to 1.38 × 10^6^ TU/mL for anti-GPRC5D. These optimized conditions were further evaluated in relation to T-cell immunophenotype and viability, enabling identification of the most efficient transduction strategy. The resulting data demonstrated an improved balance between transduction efficiency and cellular fitness (Tables S4 and S5, Supplementary Materials).

### 3.4. Immunophenotyping of T Lymphocytes After Lentiviral Transduction

**Figure 2:**
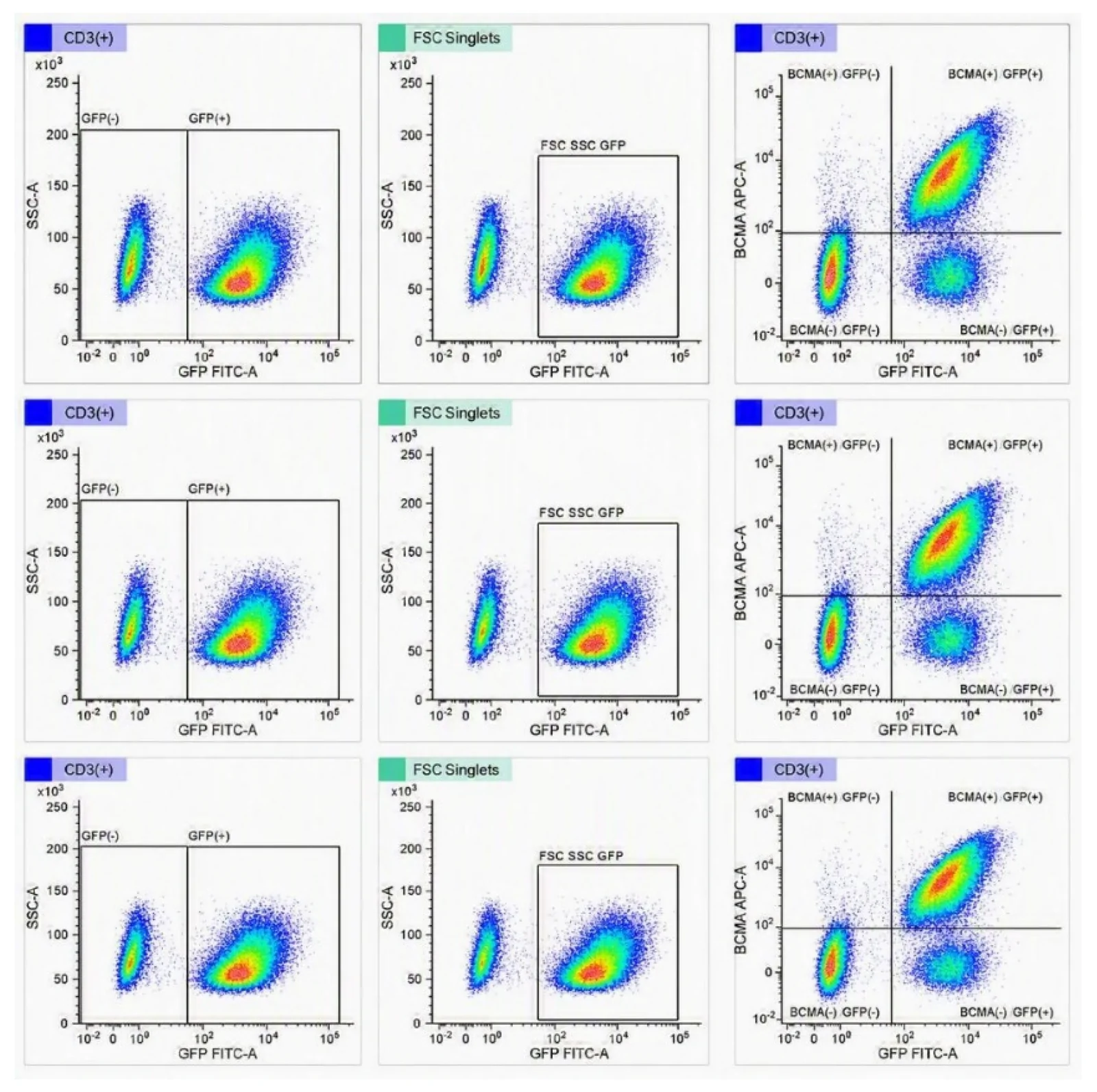
Flow cytometric analysis of GFP reporter expression in T cells transduced with the anti-GPRC5D lentiviral vector at 1.71 × 10^6^ TU/mL. Data were acquired on a BD FACS Melody and analyzed using BD Horus software.

**Figure 3:**
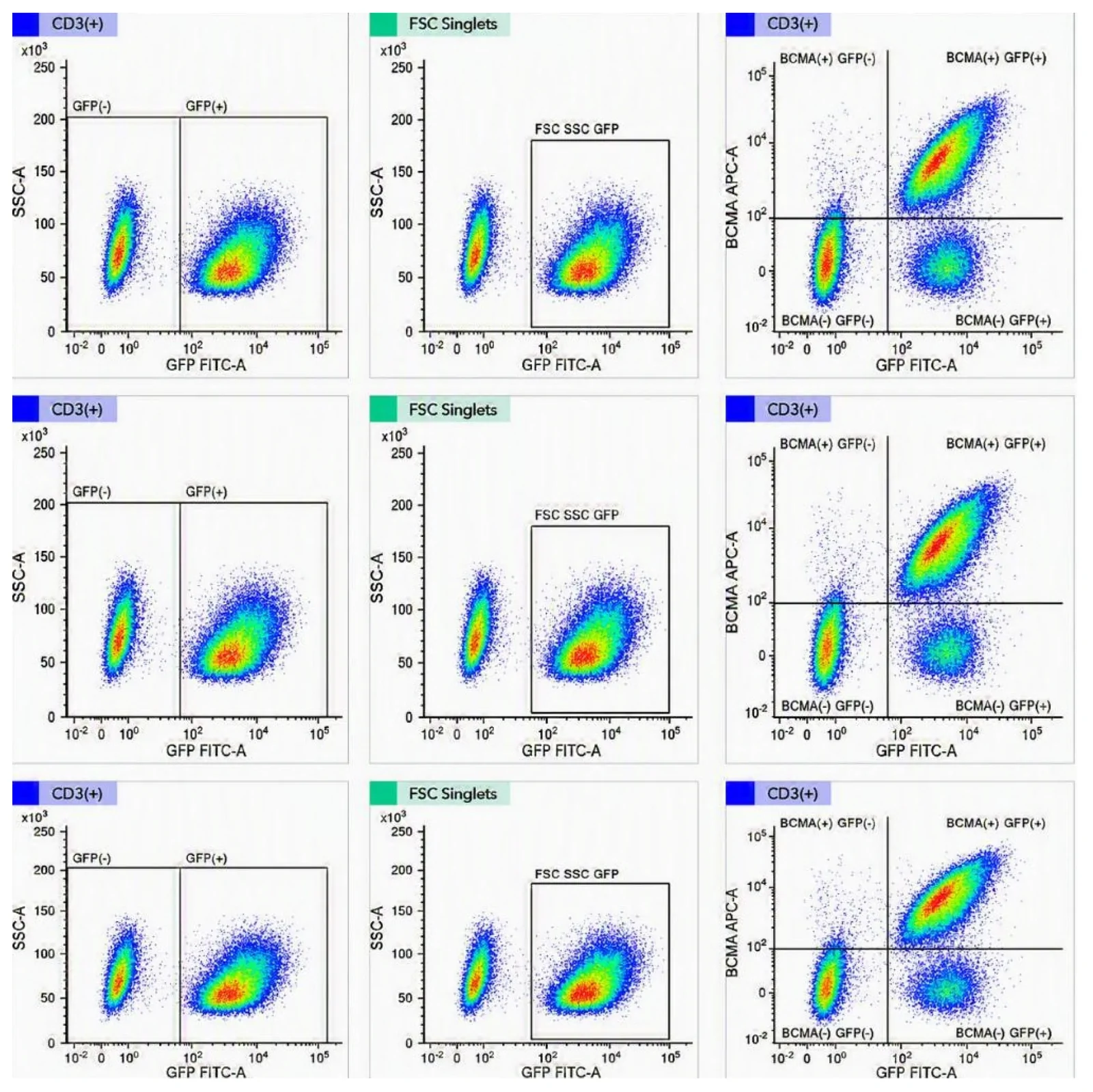
Flow cytometric analysis of GFP reporter expression and BCMA-binding populations in co-transduced T cells. Data were acquired on a BD FACS Melody and analyzed using BD Horus software.

Immunophenotyping confirmed successful generation of CAR-T cells following lentiviral transduction (Table S5). The highest BCMA-CAR transduction achieved 43.8% GFP^+^ at 2.34 × 10^6^ TU/mL; GPRC5D-CAR yielded ~13–14% GFP^+^ within the tested TU/mL range. Co-transduction experiments (1.88–3.89 × 10^6^ TU/mL for BCMA; 0.94–1.38 × 10^6^ TU/mL for GPRC5D) yielded approximately 62–66% GFP^+^ cells with a ~45–47% BCMA^+^ fraction and a GFP^+^/BCMA^−^ subset consistent with GPRC5D-CAR expression. The CD4^+^/CD8^+^ ratio ranged from 64.4% to 35.6%, respectively, with a predominance of helper T cells. Within the CD4^+^ compartment, only 5.2% of cells displayed a naïve phenotype, indicating progression toward effector differentiation. Among CD8^+^ cells, effector memory (54.6%) and central memory (34.7%) subsets predominated, suggesting a phenotype associated with enhanced proliferative capacity. Activation analysis revealed that ~98% of both CD4^+^ and CD8^+^ populations co-expressed CD69 and CD25, consistent with a highly activated state. In contrast, mono-GPRC5D CAR-T cells exhibited a lower transduction efficiency (10.3%), confirmed by GFP expression. This population was enriched for CD8^+^ T cells (83.4%) compared with CD4^+^ cells (16.6%). CD8^+^ cells were primarily composed of effector memory (41.5%), effector (25.2%), naïve (17.3%), and central memory (15.9%) subsets, indicating a heterogeneous but functionally active composition. Activation profiling showed a more moderate activation pattern in CD4^+^ cells, with CD25^+^ cells predominating and only a small fraction of CD69^+^CD25^+^ double-positive cells (13.3%), while early activation (CD69^+^CD25^−^) remained low (2.3%). Co-transduced T-cell populations were identified based on combined GFP reporter expression and BCMA-binding analysis. Double-positive populations accounted for 53.9%, while 8% of cells remained GFP single-positive. Although these findings support successful co-transduction, direct surface detection of GPRC5D-CAR expression was not performed, and GFP expression was therefore used as a surrogate marker of transduction. The CD4^+^/CD8^+^ ratio was 65% to 35%, respectively, with limited naïve CD4^+^ cells (7.5%) and a predominance of CD8^+^ effector memory cells (44.6%), followed by central memory (28.6%), effector (13.7%), and naïve (13.1%) subsets. Co-transduced T-cell populations exhibited a high activation state, with CD69^+^CD25^+^ cells comprising 93% of CD4^+^ and 89.5% of CD8^+^ populations. Across all experimental groups, cell viability remained consistently high (>96%), as confirmed by propidium iodide exclusion and trypan blue staining (Figure 2 and Figure 3).

## 4. Discussion

Early clinical studies using first-generation CAR-T cells in ovarian cancer and metastatic renal cell carcinoma demonstrated limited antitumor efficacy and poor persistence of modified T cells, prompting the development of higher-generation CAR constructs [2]. Second-generation CD19-directed CAR-T therapies, such as Tisagenlecleucel, introduced co-stimulatory domains including CD28 and 4-1BB, while newer generations incorporated additional molecules such as ICOS and OX40 to improve T-cell persistence and function [19,20]. Despite the clinical success of CAR-T therapies targeting CD19 or BCMA in hematologic malignancies, dual-antigen CAR-T products remain limited [9]. Therefore, this study focused on the development and optimization of third-generation dual-CAR-T lentiviral constructs targeting BCMA and GPRC5D in multiple myeloma. An important aspect of the presented workflow is its potential translational applicability in decentralized or academic CAR-T manufacturing settings. Current CAR-T production pipelines remain highly dependent on centralized GMP facilities, prolonged manufacturing timelines, and expensive vector production systems, creating increasing interest in simplified and scalable lentiviral manufacturing approaches suitable for early-stage development and academic-grade CAR-T generation [21]. Optimization of lentiviral production demonstrated that Lipofectamine 3000 increased transfection efficiency approximately two-fold compared with the alternative reagent when using the GFP reporter plasmid. In contrast, only minor differences were observed between the anti-BCMA and anti-GPRC5D constructs, suggesting that transfection efficiency is strongly influenced by variables including plasmid quality, vector size, DNA-to-reagent ratio, and packaging cell condition [22]. Consistent with previous reports, efficient lentiviral generation and T-cell transduction require optimization of viral titer, MOI, promoter activity, vector design, and culture conditions [23,24,25,26,27,28]. Importantly, the optimized workflow used in this study supported efficient lentiviral transduction and preservation of high T-cell viability while generating immunophenotypic profiles compatible with further CAR-T process development [29]. Although both constructs were driven by the same EF1α promoter, differences in transduction efficiency and cellular phenotype may have been influenced by the distinct co-stimulatory domains. Previous studies indicate that CD28, 4-1BB, OX40, and ICOS domains primarily affect T-cell activation, proliferation, and persistence rather than initial transduction rates [30,31]. In our study, the BCMA CAR construct containing CD28/OX40 domains was associated with higher GFP-based transduction efficiency and a more favorable expansion profile than the GPRC5D construct containing ICOS/4-1BB domains [32]. Interestingly, the magnitude of transduction improvement observed during optimization with the GFP reporter vector was not fully reproduced with the therapeutic CAR constructs. This finding suggests that reporter-gene systems may not fully predict the performance of clinically relevant lentiviral vectors. Although both CAR constructs were produced using identical manufacturing conditions and the same EF1α promoter, the GPRC5D vector demonstrated lower transduction efficiency than the BCMA construct. Because vector production parameters were standardized, manufacturing-related variability is unlikely to fully explain these differences, suggesting a potential contribution of construct-related factors such as transgene sequence composition, transcript stability, vector genome architecture, or packaging efficiency. The observed immunophenotypic differences may also have translational implications. The enrichment of effector memory CD8+ cells in the GPRC5D-transduced population could support rapid antitumor activity but may be associated with reduced persistence, whereas the more balanced memory profile observed in BCMA-CAR T cells may favor sustained immune surveillance. Several groups have recently reported tandem, bicistronic, and dual-vector BCMA/GPRC5D CAR-T platforms designed to mitigate antigen escape and improve durability of response; however, comparative manufacturing data across these approaches remain limited. Compared with tandem and bicistronic dual-targeting CAR strategies, which ensure simultaneous expression of both targeting domains within a single cell, the co-transduction approach used in this study offers a technically straightforward and flexible platform for early-stage development and optimization of dual-antigen CAR-T products, albeit at the cost of greater cellular heterogeneity. Phenotypic analysis of transduced T cells revealed a predominance of CD4+ T cells with a relatively low proportion of naïve helper cells, suggesting differentiation toward effector phenotypes. Within the CD8+ population, effector memory cells predominated, particularly in the GPRC5D CAR-T product, indicating strong early cytotoxic potential but potentially reduced long-term persistence. The observed enrichment of effector memory and central-memory subsets may be particularly relevant in multiple myeloma, where persistence and sustained immune surveillance remain major therapeutic challenges [33]. In contrast, the BCMA CAR-T construct showed higher transduction efficiency, greater CD4+ representation, and a more balanced memory phenotype, which may favor sustained immune responses. From a translational perspective, combined targeting of BCMA and GPRC5D represents a rational strategy to address antigen escape in multiple myeloma; however, functional validation will be required to determine whether the generated co-transduced populations provide improved antitumor activity [7]. Together, these findings suggest that CAR architecture and co-stimulatory domain composition may influence transduction efficiency, expansion behavior, and immunophenotypic characteristics of the generated T-cell products [34]. While GFP reporter vectors are widely used for optimization of lentiviral production workflows, their performance may not fully reflect that of therapeutically relevant constructs. In the present study, the improvement observed using the GFP reporter system was less pronounced for the BCMA- and GPRC5D-CAR vectors, suggesting that construct-specific factors, including transgene size, sequence composition, and vector architecture, may influence vector production and transduction efficiency. These findings highlight a limitation of relying exclusively on reporter systems during process development and support the need for validation using clinically relevant therapeutic constructs. Although comprehensive functional characterization was beyond the scope of the present study, preliminary experiments support the functional competence of BCMA/GPRC5D co-transduced CAR-T cells generated using the optimized manufacturing protocol, indicating that the developed workflow is suitable for the production of CAR-T cell products intended for subsequent functional and preclinical evaluation. Nevertheless, the present study did not include formal cytotoxicity assays or in vivo validation. Therefore, further studies will be required to comprehensively assess the antitumor activity, persistence, and therapeutic potential of the generated dual-targeting CAR-T cell products.

## 5. Conclusions

This study presents an optimized research-grade workflow for lentiviral vector production and T-cell transduction using CAR constructs targeting BCMA and GPRC5D. Lipofectamine 3000 outperformed TurboFectin 8.0 in HEK293T-based vector production, supporting higher viral titers and improved GFP reporter expression. The optimized protocol enabled the generation of BCMA-CAR, presumptive GPRC5D-CAR-transduced, and co-transduced T-cell populations while maintaining high cell viability and defined CD4/CD8 and memory-associated immunophenotypic profiles. Importantly, the study should be interpreted as a manufacturing and transduction optimization platform rather than a functional therapeutic validation. Notably, the lower transduction efficiency observed for the GPRC5D construct compared with the BCMA construct highlights the need for further optimization of vector design and transduction strategies in future studies. These findings provide a practical basis for the further development of dual-targeting BCMA/GPRC5D CAR-T workflows, particularly in early translational and academic process development settings.

Limitations: An important limitation of this study is the absence of direct functional characterization of the generated CAR-T cells, including antigen-specific cytotoxicity, cytokine secretion profiling, serial killing assays, exhaustion marker dynamics, and long-term persistence analyses. Consequently, the present work should primarily be interpreted as a manufacturing and transduction optimization study rather than a comprehensive therapeutic validation. Another limitation is the use of commercially available T-cell lines instead of primary donor-derived peripheral blood mononuclear cells obtained from multiple donors. Donor-to-donor variability is known to substantially influence transduction efficiency, expansion kinetics, differentiation state, and CAR-T functionality. Additionally, although the study demonstrates successful co-transduction, direct surface detection of GPRC5D-CAR expression was not performed, and GFP reporter expression was therefore used as an indirect surrogate marker of transduction. Future studies incorporating multiplex flow cytometry or single-cell approaches would help clarify the exact composition of the generated cell populations. Finally, the workflow was optimized under research-grade laboratory conditions and was not evaluated in GMP-compliant or closed-system manufacturing settings. Therefore, further process adaptation would be required before clinical-scale implementation.

Future directions: Future studies should focus on functional validation of the generated CAR-T cells using multiple myeloma models expressing BCMA and GPRC5D antigens. In particular, comparative analyses of mono-targeting versus dual-targeting CAR-T products would help determine whether the observed phenotypic differences translate into improved antitumor efficacy and persistence. Additional optimization of vector architecture, promoter selection, cytokine supplementation, and transduction enhancers may further improve manufacturing efficiency. Moreover, incorporation of single-cell transcriptomic analyses could provide deeper insight into the activation states, exhaustion profiles, and memory-associated signatures of the generated CAR-T populations. Given the increasing interest in decentralized and academic CAR-T manufacturing models, scalable and reproducible workflows such as the one presented here may contribute to lowering barriers associated with early-stage CAR-T development.

## 6. Patents

As a result of the work described in this manuscript, a patent procedure is in progress—P31935EP00/KJE.

## Figures and Tables

**Table 1:** Effect of GFP-encoding plasmid DNA transfection reagent, ratio, and 293T cell confluence on transfection efficiency. Representative values are reported are the mean of three replicates. Cq values obtained by qRT-PCR are converted into vector copy numbers using a kit-specific standard curve provided in the Lenti-X qRT-PCR Titration Kit (Takara Bio USA, Inc., San Jose, CA, USA; cat. no. 631235).

Sample	% Singlets	% Live Cells (PI−)	% Live Cells GFP+	% Dead Cells (PI+)	% Dead Cells GFP+	Number of Cells to All Events	Cq	Copy Number of the Virus
Lipofectamine 1:2 5 × 10^4^ cells/cm^2^	97.1%	89.6%	71.3%	9.8%	86.7%	18.5%	17.37	8.75 × 10^7^
Lipofectamine 1:2 7.5 × 10^4^ cells/cm^2^	97.4%	91.4%	64.3%	8%	80.4%	20.3%	16.59	1.49 × 10^8^
Lipofectamine 1:3 10 × 10^4^ cells/cm^2^	96.3%	93.6%	58.4%	5.9%	78.1%	25.3%	17.24	9.57 × 10^7^
Lipofectamine 1:2 12.5 × 10^4^ cells/cm^2^	96.4%	94.6%	57.7%	4.8%	76.4%	25.5%	17.08	1.07 × 10^8^
Turbofectin 1:2 5 × 10^4^ cells/cm^2^	96.1%	96.7%	32.3%	3.1%	54.5%	33.7%	19.56	1.98 × 10^7^
Turbofectin 1:3 10 × 10^4^ cells/cm^2^	96.4%	96%	32.4%	3.8%	49.4%	17.5%	19.5	2.06 × 10^7^

**Table 2:** Influence of the transfection reagent-to-plasmid DNA ratio, plasmid encoding anti-BCMA and anti-GPRC5D ratio and 293T cell confluency on transfection efficiency. The given values are the averages of triplicates.

Sample	% Singlets	% Live Cells (PI−)	% Live Cells GFP+	% Dead Cells (PI+)	% Dead Cells GFP+	Number of Cells to All Events	Copy Number of the Virus
Anti-GPRC5D Lipofectamine 1:3	99%	92%	99%	7.9%	98.6%	12.5%	2.43 × 10^7^
Anti-GPRC5D Turbofectin 1:3	98.9%	92.8%	96.1%	6.9%	98.1%	18.7%	2.52 × 10^6^
Anti-BCMA Lipofectamine 1:3	99%	94.1%	95.6%	5.7%	97.8%	19.1%	2.05 × 10^6^
Anti-BCMA Turbofectin 1:3	98.8%	92.9%	88.5%	6.7%	94.4%	24.6%	2.12 × 10^5^
Anti-BCMA Control Cells	98.4%	96.6%	0%	3.2%	0%	57.7%	0
Anti-GPRC5D Control Cells	96.3%	96%	0%	3.7%	0%	65.5%	0

**Table 3:** Anti-GPRC5D and anti-BCMA lentivirus DNA and p24 protein titers obtained from concentrated medium collected from transfected 293T cells.

qPCR	After Exponentiation	Copies/mL
anti-GPRC5D	1,930,112	9.65 × 10^8^
Control	1	326
anti-BCMA	1,065,024	5.33 × 10^8^
ELISA	average absorbance	
anti-GPRC5D	0.45	1.06 × 10^9^
anti-BCMA	0.33	6.91 × 10^8^

## Data Availability

The raw data supporting the conclusions of this article will be made available by the authors on request.

## Supplementary Materials

The following supporting information can be downloaded at https://www.mdpi.com/article/10.3390/cimb48070679/s1.
