# Supplementary material for: A Scalable Lentiviral Workflow for Laboratory-Scale Generation of BCMA/GPRC5D Co-Transduced CAR-T Cells in Multiple Myeloma
Source: Curr Issues Mol Biol. 2026 Jun 30;48(7):679. doi: 10.3390/cimb48070679 (PMC13407417; doi:10.3390/cimb48070679)
Supplement: Supplementary file 1 [file cimb-48-00679-s001.zip › cimb-4357116-supplementary.pdf]

A Scalable Lentiviral Workflow for Laboratory-Scale Generation of BCMA/GPRC5D Co-Transduced CAR-T Cells in Multiple Myeloma

Ewa Nowak [1,*], Emilia Morawiec [1,2], Adam Pudełko [1,3], Agnieszka Polak [1,4], Mateusz Broncel [1], Daria Matczyńska [1], Dawid Zamojski [1,5], Michał Czerwinski [1], Anna Bednarska-Czerwińska [1,6,7]

Supplementary Materials S1:

Protocol for transfection of 293T cells with lentiviral plasmid encoding GFP pLenti-EF1a-C-mGFP-P2A-Puro Lentiviral Gene Expression Vector.

Day 0: Cell Seeding

1. 5 × 10^4, 7.5 × 10^4, 10 × 10^4, 12.5 × 10^4 cells/cm2 were seeded in 100 μL complete DMEM each in 96-well plate (12 wells for Lipofectamine 3000 testing, 9 wells for TurboFectin 8.0 testing and 3 control wells for each cell density parameter).
2. Incubated at 37°C, 5% CO_2 overnight.

Day 1: Lipofectamine 3000 transfection

1. 70–90% confluency were reached at transfection day.
2. 50 μL of complete DMEM medium was removed from each well.
3. DNA/transfection reagent complexes for 12 wells were prepared.

For the ratio of 1 to 1.5:

- In a sterile tube 2.25 μL of Lipofectamine 3000 was diluted in 125 μL Opti-MEM Reduced Serum Medium (Gibco, cat. no. 31985070) and vortexed.
- In another tube 2.25 μL [1.125 μg] Lenti-vpak Lentiviral Packaging plasmid DNA, 0.75 μL [0.375 μg] GFP pLenti-EF1a-C-mGFP-P2A-Puro Lentiviral plasmid DNA and 3 μl of P3000 were diluted in 125 μL Opti-MEM Reduced Serum Medium, pipetted, not vortexed.
- Tube 1 was added to tube 2 and incubated in the dark for 20 min, RT, allowed for complex formation.

For the ratio of 1 to 2.3:

- In a sterile tube 3.5 μL of Lipofectamine 3000 was diluted in 125 μL Opti-MEM Reduced Serum Medium (Gibco, cat. no. 31985070) and vortexed.
- In another tube 2.25 μL [1.125 μg] Lenti-vpak Lentiviral Packaging plasmid DNA, 0.75 μL [0.375 μg] GFP pLenti-EF1a-C-mGFP-P2A-Puro Lentiviral plasmid DNA and 3 μL of P3000 were diluted in 125 μL Opti-MEM Reduced Serum Medium, pipetted, not vortexed.
- Tube 1 was added to tube 2 and incubated in the dark for 20 min, RT, allowed for complex formation.

For the ratio of 1 to 3:

- In a sterile tube 4.5 μL of Lipofectamine 3000 was diluted in 125 μL Opti-MEM Reduced Serum Medium (Gibco, cat. no. 31985070) and vortexed.
- In another tube 2.25 μL [1.125 μg] Lenti-vpak Lentiviral Packaging plasmid DNA, 0.75 μL [0.375 μg] GFP pLenti-EF1a-C-mGFP-P2A-Puro Lentiviral plasmid DNA and 3 μL of P3000 were diluted in 125 μL Opti-MEM Reduced Serum Medium, pipetted, not vortexed.
- Tube 1 was added to tube 2 and incubated in the dark for 20 min, RT, allowed for complex formation.

Control:

- In a sterile tube 4.5 μL of Lipofectamine 3000 was diluted in 256 μL Opti-MEM Reduced Serum Medium (Gibco, cat. no. 31985070) and vortexed.

4. DNA/lipofectamine mix in various ratios and the control were added dropwise to specific wells containing 50 μL complete DMEM medium and mixed gently.
5. After 6 hours, the medium with the transfection mix was replaced with fresh complete DMEM medium.

Day 1: TurboFectin 8.0 transfection

1. 70–90% confluency were reached at transfection day.
2. Complete DMEM medium was removed from each well and 50 μL of Opti-MEM was added.
3. DNA/transfection reagent complexes for 12 wells were prepared.

For the ratio of 1 to 2:

- In a sterile tube 1.2 μL [0.5 μg/μL] Lenti-vpak Lentiviral Packaging plasmid DNA, and 1 μL [0.5 μg/μL] GFP pLenti-EF1a-C-mGFP-P2A-Puro Lentiviral plasmid DNA were diluted in 100 μL Opti-MEM Reduced Serum Medium, pipetted.
- 4.4 μL of TurboFectin 8.0 reagent was added to the prepared DNA dilution, pipetted gently, and incubated in the dark for 30 min, RT, allowed for complex formation.

For the ratio of 1 to 3:

- In a sterile tube 1.2 μL [0.5 μg/μL] Lenti-vpak Lentiviral Packaging plasmid DNA, and 1 μL [0.5 μg/μL] GFP pLenti-EF1a-C-mGFP-P2A-Puro Lentiviral plasmid DNA were diluted in 100 μL Opti-MEM Reduced Serum Medium, pipetted.
- 6.6 μL of TurboFectin 8.0 reagent was added to the prepared DNA dilution, pipetted gently, and incubated in the dark for 30 min, RT, allowed for complex formation.

Control:

- In a sterile tube 6.6 μL of TurboFectin 8.0 reagent was diluted in 100 μL Opti-MEM, pipetted gently.

4. DNA/turbofectin mix in various ratios and the control were added dropwise to specific wells containing 50 μL of Opti-MEM medium and mixed gently.
5. After 6 hours, the medium with the transfection mix was replaced with complete DMEM medium.

Day 2: Virus Collection

- The supernatant was collected (~100 μL), stored at 4 degrees, fresh DMEM was added to transfected cells and microscopic analysis was performed.

Day 3: Virus Collection

- The second batch of supernatant was collected and combined with the first batch. The entire volume was centrifuged briefly, and filtered through 0.45 μm.
- Stored at -80°C long-term.

Supplementary Materials S2:

Protocol for transfection of 293T cells with lentiviral plasmid encoding Anti-BCMA scFv(25D2) h (CD28-OX40-CD3ζ) CART, pCDCAR1 and Anti-GPRC5D (ET150-8) h (ICOS-4-1BB-CD3ζ) CAR, pCDCAR1.

Day 0: Cell Seeding

1. 1 × 105 cells/cm2 were seeded in 500 μL complete DMEM each in 24-well plate (3 wells for anti-BCMA testing, 3 wells for anti-GPRC5D testing and 2 control wells).
2. Incubated at 37°C, 5% CO_2 overnight.

Day 1: Lipofectamine 3000 transfection Anti-BCMA

1. 90% confluency were reached at transfection day.
2. 250 μL of complete DMEM medium was removed from each well.
3. DNA/transfection reagent complexes for 3 wells were prepared.
4. In a sterile tube 3.375 μL of Lipofectamine 3000 was diluted in 187.5 μL Opti-MEM Reduced Serum Medium and vortexed.
5. In another tube 1.125 μL [0.5625 μg] Lenti-vpak Lentiviral Packaging plasmid DNA, 2.8125 μL [0.5625 μg] Anti-BCMA scFv(25D2) h (CD28-OX40-CD3ζ) CART, pCDCAR1 plasmid DNA and 4.5 μL of P3000 were diluted in 187.5 μL Opti-MEM Reduced Serum Medium, pipetted, not vortexed.
6. Tube 1 was added to tube 2 and incubated in the dark for 20 min, RT, allowed for complex formation.
7. DNA/lipofectamine mix was added dropwise to specific wells containing 250 μL complete DMEM medium and mixed gently.

8. After 6 hours, the medium with the transfection mix was replaced with fresh complete DMEM medium.

Day 1: TurboFectin 8.0 transfection

1. 90% confluency were reached at transfection day.
2. Complete DMEM medium was removed from each well and 250 μL of Opti-MEM was added.
3. DNA/transfection reagent complexes for 3 wells were prepared (ratio of 1 to 3).
4. In a sterile tube 1.8 μL [0.5 μg/μL] Lenti-vpak Lentiviral Packaging plasmids DNA, and 3.75 μL [0.2 μg/μL] Anti-BCMA scFv(25D2) h (CD28-OX40-CD3ζ) CART, pCDCAR1 plasmid DNA were diluted in 150 μL Opti-MEM Reduced Serum Medium, pipetted.
5. 4.95 μL of TurboFectin 8.0 reagent was added to the prepared DNA dilution, pipetted gently, and incubated in the dark for 30 min, RT, allowed for complex formation.
6. DNA/turbofectin mix in various ratios and the control were added dropwise to specific wells containing 250 μL of Opti-MEM medium and mixed gently.
7. After 6 hours, the medium with the transfection mix was replaced with complete DMEM medium.

For transfection of cells with Anti-GPRC5D (ET150-8) h (ICOS-4-1BB-CD3ζ) CAR, pCDCAR1 plasmid, follow the above protocol.

Day 2: Virus Collection

- The supernatant was collected (~1000 μL), stored at 4 degrees, fresh DMEM was added to transfected cells and microscopic analysis was performed.

Day 3: Virus Collection

- The second batch of supernatant was collected and combined with the first batch. The entire volume was centrifuged briefly, and filtered through 0.45 μm.
- Stored at -80°C long-term.

Supplementary Materials S3

The optimized transfection protocol with lentiviral plasmid encoding Anti-BCMA scFv(25D2) h (CD28-OX40-CD3ζ) CART, pCDCAR1 and Anti-GPRC5D (ET150-8) h (ICOS-4-1BB-CD3ζ) CAR, pCDCAR1 for large-scale virus production (according to patent application number 23461647.2). The 293T cells cultured to reached 90% confluency. The efficient ratio of Lipofectamine reagent to DNA concentration was determined experimentally (Table 1). This protocol was intended for transfection performed on a 75 cm2 culture bottle with an optimized seeding density of 1x105 cells/cm2.

1. 1984 μl Opti-MEM I Reduced Serum Medium, GlutaMAX Supplement (Gibco) with 71.4 μl Lipofectamine 3000 were vortexed.

2. 2005.8 µl Opti-MEM I Reduced Serum Medium, GlutaMAX Supplement, a 34 µl Packaging plasmids [0.5 µg/µl], 46.3 µl P3000 and 6,7 µl anti-BCMA plasmid [0.85 µg/µl] or anti-GPRC5D plasmid [0.85 µg/µl] were mixed.
3. Tube with lipofectamine were added to tube with plasmid DNA and incubated for 30 minutes at room temperature in the dark. Once nucleic acids were complexed with transfection reagents, stayed protected from the activity of nucleases and highly charged molecules.
4. The half amount (~ 5 ml) of culture medium was removed from the culture and the mixture was added to the bottle.
5. After 8 hours, the transfection medium was removed and fresh complete culture medium was added (10 ml) - the time with transfection mix was estimated experimentally and the most efficient was 8 to 10 hours, not longer. Longer time caused more visible cytotoxic effect on cells.

After 18 hours from transfection the first part of supernatant was collected and fresh DMEM + 10% FBS medium (10 ml) was added. The second part of supernatant was collected 48 hours after transfection. The collected supernatants were mixed and filter with a 0.45 µm filter.

Supplementary Materials S4

Cell labeling for flow cytometric analysis was performed as follows:

1. Collected a volume of suspension containing 1 × 106 cells into a polystyrene cytometry tube.
2. Centrifuged cells at 400 RCF for 5 minutes.
3. Resuspended the pellet in 1 mL of BD (FBS) Stain Buffer.
4. Centrifuged cells at 400 RCF for 5 minutes.
5. Resuspended the pellet in 100 µL of BD (FBS) Stain Buffer.
6. Previously prepared antibodies were added to cells suspended in 100 µL BD (FBS) Stain Buffer.
7. Incubated 60 min, RT in the dark (if using BCMA antibodies), or 30 min RT in the dark (if using the other antibodies).
8. Added 1mL of BD (FBS) Stain Buffer.
9. Centrifuged cells at 400 RCF for 5 minutes.
10. Resuspended the pellet in 300 µL of BD (FBS) Stain Buffer.
11. Added propidium iodide (if cell viability was necessary).
11. Proceeded the cytometric analysis.

The use of antibodies allowed the identification of subpopulations such as:

- BCMA and GPRC5D positive CAR-T cells (GFP+, BCMA+)
- GPRC5D positive CAR-T cells (GPF+, BCMA-)
- Helper T lymphocytes (CD3+, CD8-)

    - Naive Th cells (CD3+, CD8-, CD45RA+, CD62L+)
    - Early activated Th cells (CD3+, CD8-, CD69+, CD25-)
    - Activated Th cells (CD3+, CD8-, CD69-, CD25+)
- Cytotoxic T lymphocytes (CD3+ CD8+)
    - Naive Tc (CD3+, CD8+, CD45RA+, CD62L+)
    - Effector Tc (CD3+, CD8+, CD45RA+, CD62L-)
    - Central memory Tc (CD3+, CD8+, CD45RA-, CD62L+)
    - Effector memory Tc (CD3+, CD8+, CD45RA-, CD62L-)
    - Early activated Tc (CD3+, CD8+, CD69+, CD25-)
    - Activated Tc (CD3+, CD8+, CD69-, CD25+)

Table S1. The influence of transfection reagent to DNA of plasmid encoding GFP ratio and confluency of the 293T cells on transfection efficiency. The given values are the average of triplicates.

Sample	Number of cells to all events	% singlets	% live cells (PI-)	% live cells GFP+	% dead cells (PI+)	% dead cells GFP+	Cq	Copy number of the virus
Control cells 5×10^4 cells/cm2	42.8%	96.5%	97.5%	0.02%	2.3%	0%	29.07	3.12x104
Lipofectamine 1.5:1 5×10^4 cells/cm2	16.1%	97.7%	11.3%	65.7%	88.%	61%	18.66	3.65x107
Lipofectamine 2:1 5×10^4 cells/cm2	18.5%	97.1%	89.6%	71.3%	9.8%	86.7%	17.37	8.75x107
Lipofectamine 3:1 5×10^4 cells/cm2	11.5%	97.1%	87.2%	67.6%	12.4%	86.8%	17.96	5.86x107
Control Lipofectamine 3 5×10^4 cells/cm2	48.4%	96.1%	98.3%	0.04%	1.5%	0.1%	0	0
Turbofectin 2:1 5×10^4 cells/cm2	33.7%	96.1%	96.7%	32.3%	3.1%	54.5%	19.56	1.98x107
Turbofectin 3:1 5×10^4 cells/cm2	33.2%	95.8%	96.4%	30%	3.4%	52.4%	19.1	2.7x107
Control Turbofectin 3 5×10^4 cells/cm2	44.6%	96.5%	99.1%	0.03%	0.9%	0.3%	0	0
Control cells 7.5×10^4 cells/cm2	46.5%	95.7%	98.5%	0.02%	1.4%	0%	29.75	1.96x104
Lipofectamine 1.5:1 7.5×10^4 cells/cm2	22.2%	97%	93.6%	57.4%	5.9%	70.9%	17.74	6.81x107
Lipofectamine 2:1 7.5×10^4 cells/cm2	20.3%	97.4%	91.4%	64.3%	8%	80.4%	16.59	1.49x108
Lipofectamine 3:1 7.5×10^4 cells/cm2	23%	96.9%	93.8%	61.5%	5.9%	77.4%	18.07	5.44x107
Control Lipofectamine 3 7.5×10^4 cells/cm2	49.9%	93.8%	98%	0%	2.8%	0%	0	0
Turbofectin 2:1 7.5×10^4 cells/cm2	34.9%	95.7%	96.6%	31%	3.2%	55.5%	19.52	2.03x107

Sample	Number of cells to all events	% singlets	% live cells (PI-)	% live cells GFP+	% dead cells (PI+)	% dead cells GFP+	Cq	Copy number of the virus
Turbofectin 3:1 7.5×10^4 cells/cm2	31.9%	95.1%	96.4%	28%	3.4%	49.6%	19.09	2.73x107
Control Turbofectin 3 7.5×10^4 cells/cm2	56.8%	95.7%	99.2%	0.03%	0.8%	0%	0	0
Control cells 10×10^4 cells/cm2	27.6%	96.8%	94.1%	0%	5.3%	0%	31.93	4.48x103
Lipofectamine 1.5 :1 10×10^4 cells/cm2	27.3%	96%	92.7%	55.5%	6.6%	69.4%	16.96	1.16x108
Lipofectamine 2:1 10×10^4 cells/cm2	26.6%	96.7%	97%	53.9%	2.8%	72.7%	19.56	1.98x107
Lipofectamine 3:1 10×10^4 cells/cm2	25.3%	96.3%	93.6%	58.4%	5.9%	78.1%	17.24	9.57x107
Control Lipofectamine 3 10×10^4 cells/cm2	34%	93.7%	96.9%	0%	2.8%	0%	brak	brak
Turbofectin 2:1 10×10^4 cells/cm2	23.4%	96.7%	96.6%	23.5%	3.2%	44.2%	19.29	2.38x107
Turbofectin 3:1 10×10^4 cells/cm2	17.5%	96.4%	96%	32.4%	3.8%	49.4%	19.5	2.06x107
Control Turbofectin 3 10×10^4 cells/cm2	30.4%	95%	98.5%	0.01%	1.4%	0%	0	0
Control cells 12.5×10^4cells/cm2	24.5%	96.2%	95.5%	0%	4.2%	0%	31.64	5.45x103
Lipofectamine 1.5:1 12.5×10^4cells/cm2	24.5%	95.3%	95.7%	51.6%	4%	64.4%	17.37	8.75x107
Lipofectamine 2:1 12.5×10^4cells/cm2	25.5%	96.4%	94.6%	57.7%	4.8%	76.4%	17.08	1.07x108
Lipofectamine 3:1 12.5×10^4cells/cm2	22.2%	96%	94.7%	55.4%	4.8%	74.1%	16.68	1.4x108

Sample	Number of cells to all events	% singlets	% live cells (PI-)	% live cells GFP+	% dead cells (PI+)	% dead cells GFP+	Cq	Copy number of the virus
Control Lipofectamine 3 12.5×10^4cells/cm2	30.6%	94.3%	97.6%	0%	2.2%	0%	0	0
Turbofectin 2:1 12.5×10^4cells/cm2	22.2%	95.7%	96%	23%	3.8%	35.3%	20.12	1.35x107
Turbofectin 3:1 12.5×10^4cells/cm2	30%	96.4%	95.5%	25.4%	4.2%	40.5%	18.31	4.62x107
Control Turbofectin 3 12.5×10^4cells/cm2	33.3%	95.9%	96%	0.01%	3.7%	0%	0	0

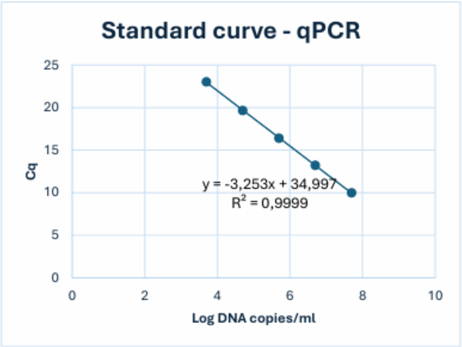

DNA copies/ml	log DNA copiers/ml	Cq
5000	3,699	23,03
50000	4,699	19,66
500000	5,699	16,40
5000000	6,699	13,21
50000000	7,699	9,99

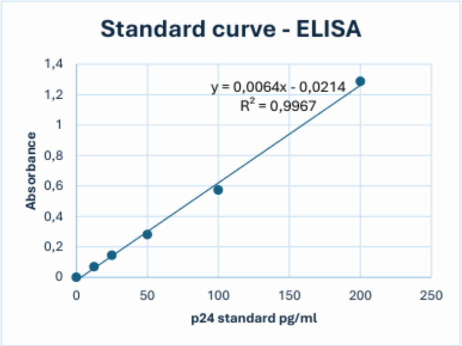

p24 pg/ml	average absorbance
12,5	0,07
25	0,15
50	0,28
100	0,57
200	1,29

Figure S1. The standard curve and the formula obtained by the Lenti-X™ qRT-PCR Titration Kit as well as by the Lenti-X p24 Rapid Titer Kit.

Table S2. Results of optimization of T lymphocyte transduction with BCMA virus. Lentiviral particle titres are reported in transduction units (TU) per ml. Mean value of GFP-positive lymphocytes 14 days after transduction.

Functional titer (TU/ml) of BCMA virus particle	GFP+
1.42x106 TU/ml	20.03%
2.34x106TU/ml	43.83%
5.33x105TU/ml	17.44%
3.55x105TU/ml	9.88%

Table S3. Results of optimization of T lymphocyte transduction with GPRC5D virus. Lentiviral particle titres are reported in transduction units (TU) per ml. Mean value of GFP-positive lymphocytes 14 days after transduction.

Functional titer (TU/ml) of GPRC5D virus particle	GFP+
1.71x106 TU/ml	13.3%
1.31x106 TU/ml	13.6%
1.08x106 TU/ml	14%
8.04x105 TU/ml	12.5%

Table S4. Immunophenotyping of dual-transduced T-cells, 14 days after transduction, including activation markers.

Functional titer (TU/ml)	GFP +	GFP+ BCMA+ dual CAR-T	GFP+ BCMA- (GPRCD5 CAR-T)	CD4+	CD8+
3.89x106TU/ml anti-BCMA and 1.38x106 TU/ml anti-GPRC5D	64.5%	45.2%	19.2%	50.5%	49.5%
1.88x106TU/ml anti-BCMA and 9.42x105 TU/ml anti-GPRC5D	65.6%	46.7%	18.8%	50.1%	49.9%

Functional titer	Th	Naive Th	Tc	Naive Tc	Cental	Effector	Effector

(TU/ml)		(CD45RA+ CD62L+)		(CD45RA+ CD62+)	memory Tc (CD45RA- CD62L+)	Tc (CD45RA + CD62L-)	memory Tc (CD45RA- CD62L-)
3.89x106TU/ml anti-BCMA and 1.38x106 TU/ml anti-GPRC5D	50.5%	1.1%	49.5%	2.2%	73.3%	0.12%	24.3%
1.88x106TU/ml anti-BCMA and 9.42x105 TU/ml anti-GPRC5D	50.1%	1.6%	49.9%	2.8%	74.3%	0.17%	22.7%

Table S5. Immunophenotyping of T cells transduced with 2.34x106TU/ml of anti-BCMA virus, 1.71x106 TU/ml of anti-GPRC5D or dual, 14 days after transduction, including activation markers.

	GFP+	GFP+ BMCA-	GFP+ BCMA+	CD4	CD8
Mono-BCMA CAR-T	60.9%	1%	59.4%	64.4%	35.6%
Mono GPRC5D CAR-T	10.3%	---		16.6%	83.4%
Dual CAR-T	62.2%	8%	53.9%	65%	35%

	Th	Naive Th (CD45RA+ CD62L+)	Tc	Naive Tc (CD45RA+ CD62+)	Cental memory Tc (CD45RA- CD62L+)	Effector Tc (CD45RA+ CD62L-)	Effector memory Tc (CD45RA- CD62L-)
Mono-BCMA CAR-T	64.4%	5.2%	35.6%	5.9%	34.7%	4.7%	54.6%
Mono-GPRC5D CAR-T	16.6%	9.1%	83.4%	17.3%	15.9%	25.2%	41.5%
Dual CAR-T	65%	7.5%	35%	13.1%	28.6%	13.7%	44.6%

	Early activated Th cells (CD69+ CD25-)	Actiaveted Th cells (CD69- CD25+)	Double positive activated Th cells (CD69+ CD25+	Early activated Tc cells (CD69+ CD25-)	Actiaveted Tc cells (CD69- CD25+)	Double positive activated Tc cells (CD69+ CD25+
Mono-BCMA CAR-T	0%	2%	98%	1%	3.6%	94.8%
Mono-GPRC5D CAR-T	2.3%	54.2%	13.3%	10%	26.2%	11.3%
Dual CAR-T	1.8%	4.6%	93%	4%	5.6%	89.5%
